# Reduction in gastrointestinal bleeding by development and implementation of a protocol for stress ulcer prophylaxis: a before-after study

**DOI:** 10.1186/s40780-015-0034-3

**Published:** 2015-12-15

**Authors:** Mai Ikemura, Shinji Nakasako, Ryutaro Seo, Takahiro Atsumi, Koichi Ariyoshi, Tohru Hashida

**Affiliations:** Department of Clinical Pharmacy, Faculty of Pharmaceutical Sciences, Kobe Gakuin University, Kobe, Hyogo Japan; Department of Pharmacy, Kobe City Medical Center General Hospital, Kobe, Hyogo Japan; Emergency Department, Kobe City Medical Center General Hospital, Kobe, Hyogo Japan

**Keywords:** Stress ulcer prophylaxis, Intensive care unit, Clinically important bleeding, Protocol

## Abstract

**Background:**

The implementation of a protocol has been associated with improvements in the processes of care in clinical settings. Although stress ulcer prophylaxis is recommended for critically ill patients at high risk, there is currently no consensus on its use. Therefore, we herein developed a protocol for stress ulcer prophylaxis, and evaluated therapeutic outcomes in a before-after study.

**Methods:**

The protocol was developed by considering the effectiveness, disadvantages (including adverse events) and cost of each agent based on previous findings. Patients who were admitted to the 8-bed emergency intensive care unit (ICU) of our hospital for more than 24 h during the year before and after implementation of the study were eligible. Each investigation item was evaluated retrospectively.

**Results:**

There were 211 and 238 study patients before and after implementation of the protocol, respectively. The baseline characteristics of patients on/during ICU admission were similar in the two groups. The proportion of medicated patients was 79.6 % before and 84.5 % after protocol implementation. Before implementation of the protocol, 4.3 % of patients developed clinically important gastrointestinal bleeding, and this incidence decreased significantly to 0.8 % after its implementation (*P* = 0.019). The frequency at which medication was discontinued due to adverse events was slightly lower after implementation of the protocol. No significant differences were observed in the costs of stress ulcer prophylactic agents or mortality in the ICU.

**Conclusions:**

The results of the present study indicated that the development and implementation of a protocol for stress ulcer prophylaxis, for which there are currently no criteria, improved a main outcome, clinically important gastrointestinal bleeding.

## Background

Stress ulcers are superficial lesions generally, but not exclusively involving the mucosal layer of the stomach, and commonly occur in critically ill patients [1]. Most critically ill patients have endoscopically detectable mucosal erosion and subepithelial hemorrhage within 24 h of admission to an intensive care unit (ICU) [2]. The frequency of clinically important gastrointestinal (GI) bleeding in patients not receiving prophylaxis reportedly ranges from 0.1 to 39 % [1]. The mortality rate is 48.5 % in ICU patients with clinically important bleeding, in contrast to 9.1 % in those without bleeding [3]. Therefore, it has been recommended that stress ulcer prophylaxis should be implemented in patients at high risk.

Approaches to stress ulcer prophylaxis include medication with pharmacological agents and enteral nutrition [1, 2, 4–11]. Agents known to provide stress ulcer prophylaxis include antacids, prostanoids, sucralfate, histamine-2 receptor antagonists (H_2_RAs) and proton pump inhibitors (PPIs). The efficacy and safety of H_2_RAs and PPIs in particular have been investigated in recent studies [5–9]. In some studies, the effects of PPIs and H_2_RAs were found to be similar, whereas others indicated that PPIs decreased the risk of GI bleeding more than H_2_RAs [6–8]. A recent study suggested that PPIs were associated with a greater risk of GI hemorrhage, pneumonia and *Clostridium difficile* infection than H_2_RAs in mechanically ventilated patients [12]. Therefore, both agents have advantages and disadvantages in clinical settings [5, 6].

Although various approaches to stress ulcer prophylaxis have been reported, there is limited evidence for and no consensus on their efficacy and safety. Few studies have proposed and examined criteria for selecting stress ulcer prophylactic agents. Since critically ill patients characteristically require various therapies, the absence of a therapeutic strategy potentially leads to inappropriate medication, which may have a negative impact on the process of care. An appropriate approach to stress ulcer prophylaxis based on the clinical characteristics of the patient, which are diverse and may vary from hour to hour, is considered necessary. The implementation of protocols has been associated with improvements in the processes of care in clinical settings [13]. Therefore, the development of a protocol for stress ulcer prophylaxis may improve the process of care in critically ill patients. In the present study, we devised a protocol for stress ulcer prophylaxis, and evaluated therapeutic outcomes in the ICU before and after its implementation.

## Methods

### Development and implementation of a protocol for stress ulcer prophylaxis

**Fig. 1:**
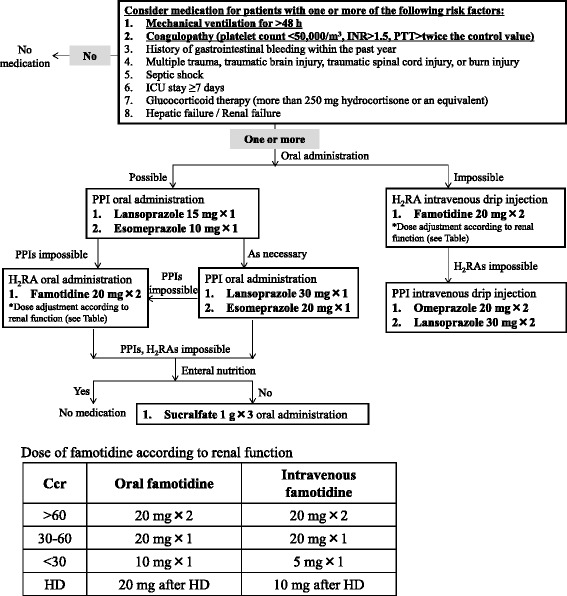
Protocol for stress ulcer prophylaxis in ICU patients. The upper panel shows the recommended procedure according to risk factors for stress ulcer prophylaxis. In patients with one or more of the listed factors, medication was considered according to the flowchart. A patient with risk factors 1 or 2 almost always received medication. When it was not possible to administer a particular agent or a patient’s condition had changed, including the possibility of oral administration, another agent was administered according to the flowchart. Recommended adjustments to the dosage of famotidine according to renal function are shown in the lower panel. Ccr, creatinine clearance; HD, hemodialysis; INR, international normalized ratio; PTT, partial thromboplastin time

A protocol was developed by intensive care specialists and clinical pharmacists, who considered the effectiveness of stress ulcer prophylaxis, adverse effects and interactions and cost of each agent, with data being drawn from published studies and Japanese drug package inserts (Fig. 1). Risk factors were determined as reported previously [1–4, 6]. Medical care was mainly based on the resultant protocol. However, where necessary, physicians were allowed the flexibility to individualize medication according to a particular patient’s characteristics, including the generation of GI bleeding and continuation of antiulcerogenic agents that were being taken prior to ICU admission.

The protocol was implemented from January 2013 for patients who fit the eligibility criteria. Intensive care physicians generally prescribed the agents specified by the protocol. In addition, pharmacists checked the patients’ conditions and medications nearly every day, and proposed changes to the physicians when the medications were not in accordance with the protocol.

### Design, setting and participants

This was a retrospective observational before-after study. Patients who were admitted to the 8-bed emergency ICU in Kobe City Medical Center General Hospital, a 700-bed general hospital, between January and December 2012 (before implementation of the protocol) or between January and December 2013 (after its implementation), were enrolled. Patients were excluded if they were younger than 20 years, had GI bleeding on ICU admission, or were discharged within 24 h of admission. Although study patients admitted to the ICU for less than 24 h were ineligible for this study, the protocol was also used to select their treatment.

### Outcome measures

Baseline characteristics, including sex, age, the presence or absence of intubation, coagulopathy, trauma and burns on/during ICU admission, medication status and outcomes were evaluated. The medication status included medication or not, the types and number of agents used for stress ulcer prophylaxis, dosages, duration of administration, adverse events and costs during the ICU stay. Medicated patients were defined as those who received one or more of the following stress ulcer prophylactic agents: intravenous lansoprazole, omeprazole, cimetidine, famotidine and ranitidine; and oral esomeprazole, lansoprazole, omeprazole, rabeprazole, famotidine, ranitidine and sucralfate. Costs included those of the agents themselves, but not of the devices used to administer the medication, nutrition or treatment of adverse effects caused by the agents. Prices were calculated in Japanese yen. Adverse events caused by stress ulcer prophylactic agents were evaluated from the medical records. The studied outcomes of application of the protocol included the number of patients with GI bleeding and their baseline characteristics, duration of stay and mortality in the ICU. Clinically important bleeding was defined as reported by Krag et al. [5]. Each investigation item was evaluated retrospectively using the electronic health record system.

### Statistical analysis

Differences before and after implementation of the protocol were evaluated statistically by the two-sample *χ*^2^ test of proportions (baseline characteristics including sex, number of intubated patients, patients with coagulopathy, trauma and burns, number of patients who received stress ulcer prophylactic agents, clinically important GI bleeding and mortality in the ICU), or the Mann-Whitney *U* test (age, medicated days, total cost of stress ulcer prophylactic agents, and duration of the ICU stay). Significance was set at *P* < 0.05.

### Ethics

This study was approved by the Institutional Review Board of Kobe City Medical Center General Hospital and the Board waived the need for patients’ consent (No. 1303-jn4).

## Results

In this study, 211 and 238 patients were eligible before and after introduction of the protocol for stress ulcer prophylaxis, respectively. Their baseline characteristics on/during ICU admission are shown in Table 1. Distribution of sex and of diagnosis and treatment department, and age were similar in the two groups. No significant differences were observed in the number of intubated patients or patients with coagulopathy, trauma or burns. The distribution of diagnoses and treatments was also similar.

**Table 1:** Baseline characteristics on/during ICU admission

		Before implementation of the protocol (*n* = 211)		After implementation of the protocol (*n* = 238)		*P* value
Sex	Male	132	(62.6 %)	157	(66.0 %)	0.452
	Female	79	(37.4 %)	81	(34.0 %)	
Age (years)^a^		70	(21–98)	72	(22–94)	0.269
Diagnosis and treatment department	Medical	110	(52.1 %)	122	(51.3 %)	0.974
	Surgery	40	(19.0 %)	47	(19.7 %)	
	Others	61	(28.9 %)	69	(29.0 %)	
Intubation		96	(45.5 %)	101	(42.4 %)	0.514
Coagulopathy		91	(43.1 %)	106	(44.5 %)	0.764
Trauma		44	(20.9 %)	38	(16.0 %)	0.181
Burns		8	(3.8 %)	5	(2.1 %)	0.286

^a^Values are presented as the median (range)

The only pharmacological agents administered for stress ulcer prophylaxis were PPIs and famotidine; ranitidine, sucralfate; the other types of stress ulcer prophylactic agents were not chosen. The proportion of medicated patients after introduction of the protocol was slightly higher than that of before the introduction, 84.5 and 79.6 %, respectively (Table 2). The frequency of choice of particular stress ulcer prophylactic agents was similar; however, oral lansoprazole was used more frequently and oral famotidine less frequently after implementation of the protocol. Furthermore, the dosages differed. For example, the proportion of patients who received 15 mg of lansoprazole increased (from 31.5 to 52.7 %), whereas the proportion who received 30 mg decreased (from 14.9 to 7.5 %). Before introduction of the protocol, 20 mg famotidine was administered to most patients, both orally and intravenously and the dosage was not always adjusted for renal function. Consequently, the proportion of patients receiving 40 mg was higher after introduction of the protocol. Discontinuation of the medication because of definite or suspected adverse events occurred less frequently after introduction of the protocol, decreasing from 6.6 to 3.8 %. The duration of administration and costs of stress ulcer prophylactic agents were similar in the two groups of patients (Table 2).

**Table 2:** Variables related to the use of stress ulcer prophylactic agents

	Before implementation of the protocol (*n* = 211)		After implementation of the protocol (*n* = 238)		*P* value
Medicated patients	168	(79.6 %)	201	(84.5 %)	0.182
Duration of administration (days)^a^	3.0	(0–36)	2.5	(0–46)	0.586
Cost (yen)^a^	538	(0–10,198)	536	(0–8,404)	0.573

^a^Values are presented as the median (range)

Table 3 summarized the outcomes of implementation of the protocol for stress ulcer prophylaxis. Mortality and stay in the ICU were similar in the two groups. The number of patients with clinically important GI bleeding significantly decreased after introduction of the protocol (from 4.3 to 0.8 %; *P* < 0.05). Before its introduction, 5 out of 9 patients with GI bleeding had not received stress ulcer prophylactic agents even though they were at high risk. PPIs and H_2_RAs were both contraindicated in 3 of these patients because of their adverse events. Furthermore, 3 patients received omeprazole or rabeprazole via a gastric tube before introduction of the protocol. However, this did not occur after introduction of the protocol, when most patients received the medication specified by the protocol. Exceptions included continuation of an antiulcerogenic agent that a patient had been taking prior to ICU admission, and the administration of no medication to patients receiving enteral nutrition. Among the patients with clinically important GI bleeding, 2 and 0 patients died in the ICU before and after the implementation of the protocol, respectively. Their direct cause of death was not GI bleeding.

**Table 3:** Outcomes before and after implementation of the protocol

	Before implementation of the protocol (*N* = 211)		After implementation of the protocol (*N* = 238)		*P* value
Clinically important bleeding	9	(4.3 %)	2	(0.8 %)	0.019
Mortality in ICU	19	(9.0 %)	22	(9.2 %)	0.930
ICU stay (days)^a^	5.0	(2–59)	4.0	(2–49)	0.195

^a^Values are presented as the median (range)

## Discussion

Previous studies demonstrated that protocols improved the processes of care [13]. Although recent studies provided recommendations, few protocols for stress ulcer prophylaxis that detail agents and dosages have been published [9, 14]. Therefore, we developed the protocol presented herein to provide criteria for stress ulcer prophylaxis, including risk factors, agents, dosages and routes, based on previous findings (Fig. 1). Confirmation by clinicians and pharmacists prevented the use of unnecessary medication and omission of medication. As a result, more patients were medicated after implementation of the protocol despite similar baseline characteristics on/during ICU admission before and after implementation (Tables 1 and 2). This is because some patients did not receive necessary medication for stress ulcer prophylaxis because of the absence of a protocol. Therefore, implementation of the protocol was expected to augment the processes of care in critically ill patients.

The duration and cost of medications for stress ulcer prophylaxis were similar before and after introduction of the protocol (Table 2). In some circumstances the duration and cost decreased; for example, fewer patients received the agents as therapy for GI bleeding, not as prophylaxis, after implementation of the protocol. Other situations increased the duration and cost, such as when fewer patients at high risk of stress ulcers did not receive medication after implementation. Furthermore, the cost was decreased by earlier switching to oral administration. Conversely, standardization of the dosage of H_2_RAs based on renal function increased the average dosage.

Before introduction of the protocol, 4.3 % of patients had clinically important GI bleeding (Table 3), which was consistent with previously reported rates [8]. This finding indicated that the medical care provided prior to protocol implementation was not substandard. After introduction of the protocol, significantly fewer patients had clinically important GI bleeding (0.8 %) (Table 3), an improvement we attributed to the implementation of the protocol. Before introduction of the protocol, some patients with GI bleeding had not received stress ulcer prophylaxis even though they were at high risk, partly because of adverse events and also because there were no clear criteria for selecting appropriate prophylactic agents. The protocol presented here includes multiple strategies to cover situations in which it is not possible to continue the administration of a particular agent. Furthermore, the protocol recommends minimum effective dosages, which may decrease the frequency of adverse events. Since adverse events occurred less frequently, medication was discontinued less often, a possible reason for the lower incidence of GI bleeding. Prior to introduction of the protocol, some patients with GI bleeding received agents that cannot be administered via a gastric tube, including omeprazole and rabeprazole. These medications may have been ineffective for stress ulcer prophylaxis. The protocol did not include these agents owing to risk management considerations; therefore, they were rarely selected after its introduction.

While the number of patients with clinically important GI bleeding was decreased after the implementation of the protocol, mortality in ICU was similar in two groups (Table 3). The mortality rate is known to be dramatically increased in patients with clinically important GI bleeding [3]. Generally, a number of fatalities might be decreased, as a number of patients with clinically important GI bleeding are decreased. In this study, few patients with clinically important GI bleeding died in ICU. Therefore, mortality in ICU was not affected by the frequency of clinically important GI bleeding.

There were some limitations in our evaluation of the protocol. Although pneumonia is known to be one of the major adverse events associated with stress ulcer prophylactic agents, we did not evaluate its frequency because many patients had pulmonary disease on ICU admission. Previous studies suggested that acid-suppressive agents increased the risk of pneumonia [15, 16], and that this may be mediated by the growth of gastric flora with increasing pH [17]. In the present study, mortality and the ICU stay were similar before and after introduction of the protocol, suggesting that there was no increase in the frequency of pneumonia.

## Conclusions

The use of our protocol for stress ulcer prophylaxis, which was designed based on previous findings, resulted in a decrease in the frequency of clinically important GI bleeding in critically ill patients. These results indicated that the development and implementation of a protocol for stress ulcer prophylaxis, for which there are currently no criteria, might improve therapeutic outcomes.

## Abbreviations

Ccr: creatinine clearance
GI: gastrointestinal
H_2_RA: histamine-2 receptor antagonist
HD: hemodialysis
ICU: intensive care unit
INR: international normalized ratio
PPI: proton pump inhibitor
PPT: partial thromboplastin time

## References

[1] ASHP therapeutic guidelines on stress ulcer prophylaxis. Am J Health Syst Pharm. 1999;56:347–379. 10.1093/ajhp/56.4.347 10690219

[2] Mutlu GM, Mutlu EA, Factor P (2001). GI complications in patients receiving mechanical ventilation. Chest.

[3] Cook DJ, Fuller HD, Guyatt GH, Marshall JC, Leasa D, Hall R (1994). Risk factors for gastrointestinal bleeding in critically ill patients. Canadian Critical Trials Group. N Engl J Med.

[4] Tryba M, Cook D (1997). Current guidelines on stress ulcer prophylaxis. Drugs.

[5] Krag M, Perner A, Wetterslev J, Moller MH (2013). Stress ulcer prophylaxis in the intensive care unit: is it indicated? A topical systematic review. Acta Anaesthesiol Scand.

[6] Mohebbi L, Hesch K (2009). Stress ulcer prophylaxis in the intensive care unit. Proc (Baylor Univ Med Cent).

[7] Lin PC, Chang CH, Hsu PI, Tseng PL, Huang YB (2010). The efficacy and safety of proton pump inhibitors vs histamine-2 receptor antagonists for stress ulcer bleeding prophylaxis among critical care patients: a meta-analysis. Crit Care Med.

[8] Alhazzani W, Alenezi F, Jaeschke RZ, Moayyedi P, Cook DJ (2013). Proton pump inhibitors versus histamine 2 receptor antagonists for stress ulcer prophylaxis in critically ill patients: a systematic review and meta-analysis. Crit Care Med.

[9] Alhazzani W, Alshahrani M, Moayyedi P, Jaeschke R (2012). Stress ulcer prophylaxis in critically ill patients: review of the evidence. Pol Arch Med Wewn.

[10] Cook D, Heyland D, Griffith L, Cook R, Marshall J, Pagliarello J (1999). Risk factors for clinically important upper gastrointestinal bleeding in patients requiring mechanical ventilation. Canadian Critical Care Trials Group. Crit Care Med.

[11] Marik PE, Vasu T, Hirani A, Pachinburavan M (2010). Stress ulcer prophylaxis in the new millennium: systematic review and meta-analysis. Crit Care Med.

[12] MacLaren R, Reynolds PM, Allen RR (2014). Histamine-2 receptor antagonists vs proton pump inhibitors on gastrointestinal tract hemorrhage and infectious complications in the intensive care unit. JAMA Intern Med.

[13] Sinuff T, Muscedere J, Adhikari NK, Stelfox HT, Dodek P, Heyland DK (2013). Knowledge translation interventions for critically ill patients: a systematic review. Crit Care Med.

[14] Dellinger RP, Levy MM, Rhodes A, Annane D, Gerlach H, Opal SM (2013). Surviving Sepsis Campaign Guidelines Committee including the Pediatric Subgroup. Surviving Sepsis Campaign: international guidelines for management of severe sepsis and septic shock: 2012. Crit Care Med.

[15] Laheij RJ, Sturkenboom MC, Hassing RJ, Dieleman J, Stricker BH, Jansen JB (2004). Risk of community-acquired pneumonia and use of gastric acid-suppressive drugs. JAMA.

[16] Herzig SJ, Howell MD, Nqo LH, Marcantonio ER (2009). Acid-suppressive medication use and the risk for hospital-acquired pneumonia. JAMA.

[17] Gray JD, Shiner M (1967). Influence of gastric pH on gastric and jejunal flora. Gut.

